# A Pathogenic Homozygous Mutation in The Pleckstrin Homology Domain of *RASA1* Is Responsible for Familial Tricuspid Atresia in An Iranian Consanguineous Family

**DOI:** 10.22074/cellj.2019.5734

**Published:** 2018-11-18

**Authors:** Ahoura Nozari, Ehsan Aghaei-Moghadam, Aliakbar Zeinaloo, Afagh Alavi, Saghar Ghasemi Firouzabdi, Shohre Minaee, Marzieh Eskandari Hesari, Farkhondeh Behjati

**Affiliations:** 1 Genetics Research Center, University of Social Welfare and Rehabilitation Sciences, Tehran, Iran; 2 Department of Pediatrics Cardiology, Faculty of Medicine, Tehran University of Medical Sciences, Tehran, Iran

**Keywords:** Pleckstrin Homology Domain, *RASA1*, Tricuspid Atresia, Whole Exome Sequencing

## Abstract

**Objective:**

Tricuspid atresia (TA) is a rare life-threatening form of congenital heart defect (CHD). The genetic mechanisms underlying TA are not clearly understood. According to previous studies, the endocardial cushioning event, as the primary sign of cardiac valvulogenesis, is governed by several overlapping signaling pathways including Ras/ ERK pathway. RASA1, a regulator of cardiovascular development, is involved in this pathway and its haploinsufficiency (due to heterozygous mutations) has been identified as the underlying etiology of the autosomal dominant capillary malformation/arteriovenous malformation (CM/AVM).

**Materials and Methods:**

In this prospective study, we used whole exome sequencing (WES) followed by serial bioinformatics filtering steps for two siblings with TA and early onset CM. Their parents were consanguineous which had a history of recurrent abortions. Patients were carefully assessed to exclude extra-cardiac anomalies.

**Results:**

We identified a homozygous RASA1 germline mutation, c.1583A>G (p.Tyr528Cys) in the family. This mutation lies in the pleckstrin homology (PH) domain of the gene. The parents who were heterozygous for this variant displayed CM.

**Conclusion:**

This is the first study reporting an adverse phenotypic outcome of a *RASA1* homozygous mutation. Here, we propose that the phenotypic consequence of the homozygous *RASA1* p.Tyr528Cys mutation is more serious than the heterozygous type. This could be responsible for the TA pathogenesis in our patients. We strongly suggest that parents with CM/AVM should be investigated for *RASA1* heterozygous mutations. Prenatal diagnosis and fetal echocardiography should also be carried out in the event of pregnancy in heterozygous parents.

## Introduction

**Fig.1:**
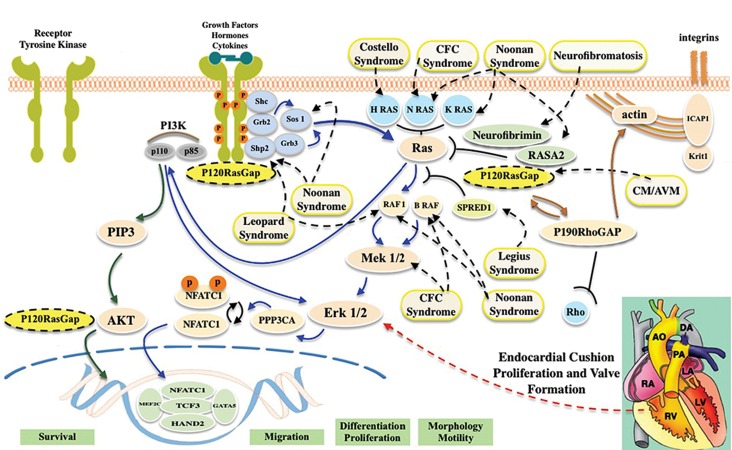
The Ras/ERK signal transduction pathway with emphasis on p120-RasGAP interactions. By the binding of growth factors, cytokines or hormones to tyrosinekinase receptors (TKR), cross phosphorylation of tyrosine residues occurs and following their dimerization, the Ras/ERK pathway is activated. RasGap proteinsincluding p120-RasGAP, neurofibromin (NF1) and RAS-P21 protein activator 2 (RASA2) are able to downregulate Ras signaling by the conversion of the active formof Ras to its inactive form (GTP-bound and GDP-bound respectively) (5, 14). P120-RasGAP, in a Ras-dependent manner, binds to phosphorylated TKRs. The Ras/ ERK pathway (shown by blue arrows) leads to differentiation, proliferation and migration and is involved in the development of heart valve. The inherited disordersof this pathway including NF1, Legius syndrome, Noonan, Cardiofaciocutaneous (CFC) syndrome, Costello, LEOPARD, and Capillary Malformation/ArteriovenousMalformation (CM/AVM) are indicated. Ras-independent function of *RASA1* (shown by brown arrows) is promoted via the interaction between p120-RasGAP andp190 RhoGAP with the latter acting as a GAP for the Ras superfamily protein Rho (15, 16). P120-RasGAP also has the ability to bind phosphoinositides. The PI3K/ AKT pathway (shown by green arrows) leads to cell survival, which protects cells against apoptosis (1, 15).

Cardiac valvulogenesis is known as an embryogenic evolutionary conserved mechanism in all vertebrates (1). Heart valve formation is described by the primary formation of endocardial cushions (ECs) in the atrioventricular canal and outflow tract, which starts at embryonic day (E) E31-E35 in human and E9.5 in mouse (2, 3). During the complex endocardial cushioning event, endothelial-mesenchymal transition occurs in a subgroup of endothelial cells and the atrioventricular canal including the mitral and tricuspid valves will appear (4). This critical stage is governed by overlapping signaling pathways including VEGF, NFATc1, Notch, Wnt/beta-catenin, BMP/TGF-beta, ErbB, EGF and Ras/ERK (MAPK) pathways (2, 4-6). The interactions among these signaling pathways and their relative timing are proposed as a signaling network model for valvulogenesis (Fig .1) (4). 

**Fig.2:**
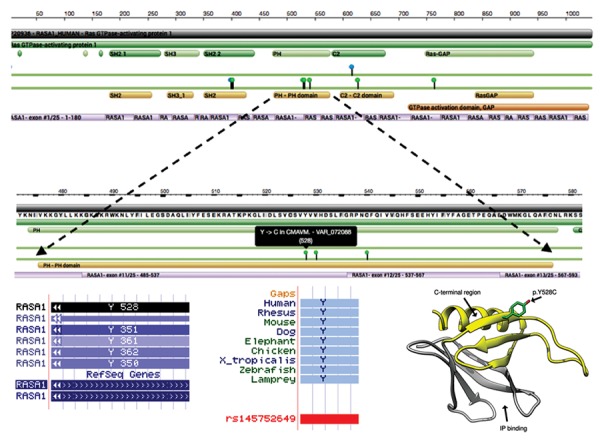
P120-RasGAP protein structure. A. P120-RasGAP is a protein with 1047 aminoacid residues with the N-terminus containing SH_2_ and SH_3_ domains, and the central region containing a PH domain and a CALB/C2 domain. The C-terminus has the RasGAP domain. The position of the p.Tyr528Cys *RASA1* missense mutation in the PH domain has been indicated (https://www.rcsb.org/), B. Position of the tyrosine residue in *RASA1* transcripts (http://genome. ucsc.edu), C. The tyrosine residue is strongly conserved among the species (http://genome.ucsc.edu), and D. The position of the p.Tyr528Cys *RASA1* missense mutation in the three-dimensional model (secondary structure) of the PH domain is shown. The p120-RasGAP PH domain comprises seven antiparallel beta-sheets, which is closed at one end by a C-terminal alpha-helix. The Tyr528 side chain is exposed in the C-terminal region, neighboring to the C-terminal alpha-helix of the PH domain (the C-terminal region of the domain and the inositol-phosphate (IP)-binding site are also shown) (12).

Numerous gene disruptions related to these pathways have now been revealed to influence valve phenotypes (7). Tricuspid atresia (TA, MIM#605067), with a prevalence of 1/25000 at live birth, is an infrequent form of valvular congenital heart defect (CHD) commonly associated with poor prognosis (1, 8, 9). Some studies have reported familial occurrences of TA (10-12). However, the genetic mechanisms underlying TA remain unclear. In this study, we used whole exome sequencing (WES) as a powerful method for detecting the genetic aetiology of a heterogeneous disease such as CHD (1, 13), and found a germline *‘homozygous’* missense mutation c.1583A>G p.(Tyr528Cys) in the pleckstrin homology (PH) domain of *RASA1* (Fig .2) in a consanguineous Iranian family.

## Materials and Methods

**Fig.3:**
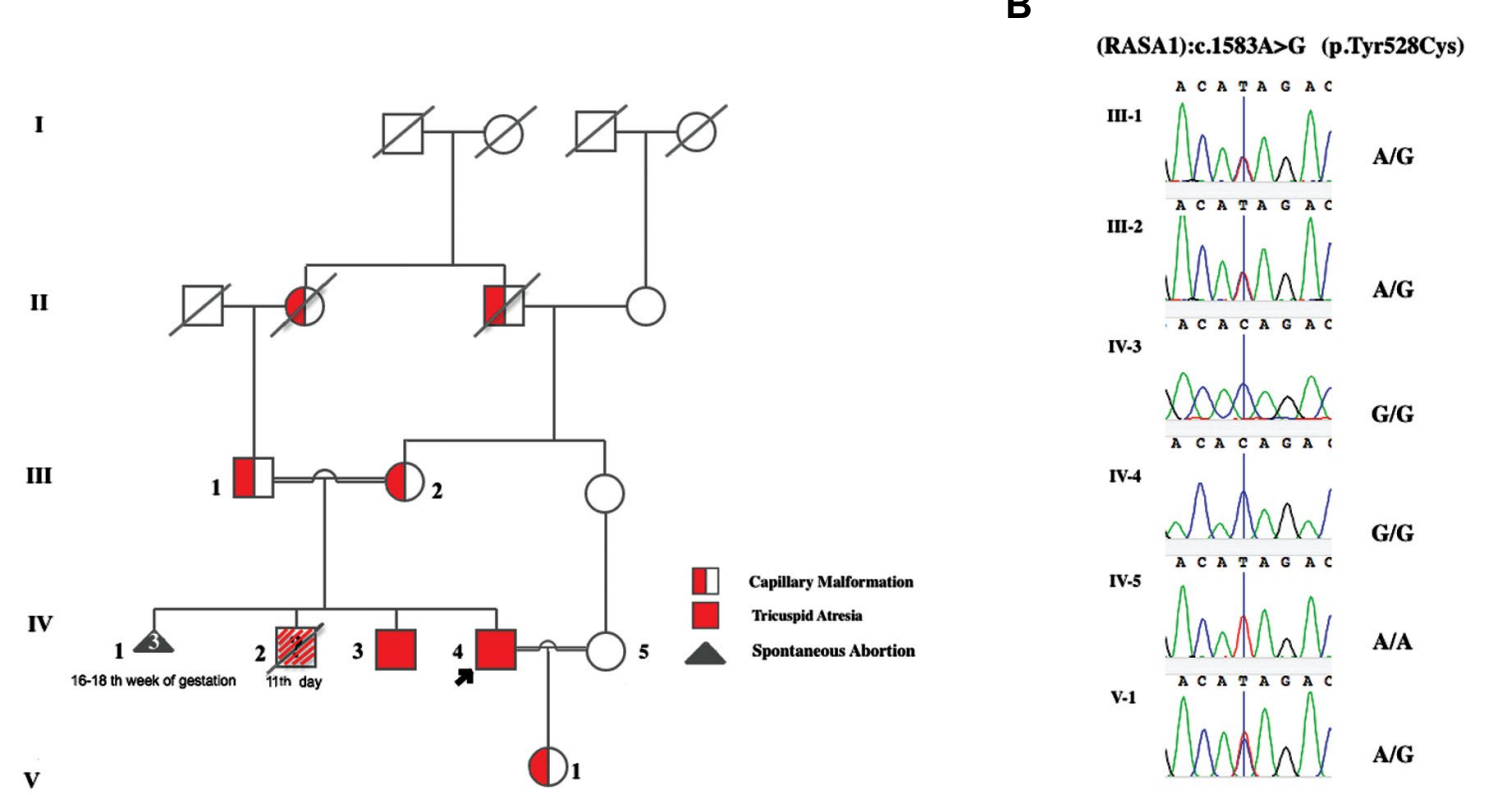
The family pedigree and chromatogram results of *RASA1*:c.1583A>G p.(Tyr528Cys). A. The pedigree displays an autosomal dominant pattern of inheritance and B. The Sanger sequencing validation for all the family members. Individual IV-2 died due to cardiac anomalies, TA was not investigated.

In this prospective study, a consanguineous family, in which the parents were first cousins, were referred to the Pediatric Cardiology and Neonatal Intensive Care Unit of Tehran Children Medical Center for prenatal ultrasound screening of CHD for high risk families. The fetus’ father (proband) as well as her paternal uncle were already diagnosed with TA (currently at ages 32 and 28 years respectively). The affected siblings with TA were born to healthy consanguineous parents which had a history of three pregnancy losses in 16-18 weeks of gestation. In this family, there was also one infant who had died at day 11 after birth with an unknown heart malformation (Fig .3). Although prenatal ultrasound screening of CHD for the proband’s fetus appeared normal, this family was interested in determining the genetic aetiology underlying CHD in their family. A signed informed consent form was taken from all participants after being informed of the aim of the research study. This research study was approved by the Ethics Committee of the University of Social Welfare and Rehabilitations Sciences of Tehran, Iran (IR.USWR.REC.1395.132).

## Cytogenetics and Fluorescent in Situ Hybridization analysis

For classical cytogenetics analysis, 5 ml venous blood samples-collected in heparinized tubes-were handled for cell culture and harvesting following standard techniques. High-resolution G-banded lymphocyte culture (520 resolution) was carefully analyzed to exclude chromosomal abnormality in patients. 

Fluorescent in Situ Hybridization (FISH) analysis was carried out on a suspension of metaphase and interphase cells using KreatechTM KBI-40103 DiGeorge HIRA (22q11)/22q13 (SHANK3) probes, according to the manufacturer’s procedure, to exclude the 22q11.2 microdeleion. 

## Exome sequencing

Genomic DNA was extracted from 5 ml venous blood collected in EDTA-containing tubes using the standard salting-out method. Approximately 50 ng of genomic DNA was obtained from the proband and prepared for WES with the Exome Enrichment Kit and Agilent’s SureSelect Human All Exon V6 capture probes, and run on an Illumina HiSeq 4000 platform yielding an average read depth of 100x. Sequence alignment and variant calling for the targeted platform were made against the human reference genome GRCh37/hg19 build and wANNOVAR (http://wannovar.wglab.org/) was used for variant annotation.

## Bioinformatic analysis

Several steps were taken to prioritize the entire set of high-quality variants. Briefly, variants in intergenic, down/up-stream, intronic, and UTR regions along with synonymous variants were excluded. Based on the hypothesis that the causative mutation for the disease in the siblings is rare, variants with unreported and reported minor allele frequency (MAF)=0.01 were considered in genomic variation databases including exac, (http://exac.broadinstitute.org/), the 1000 Genomes project (www.1000genomes.org), genomAD browser (http://gnomad.broadinstitute.org) and NHLBI Exome Sequencing Project (ESP) (http://evs.gs.washington.edu/ EVS/). Moreover, variants observed in the exomes of 100 unrelated healthy Iranians or Iranians affected with non- cardiovascular diseases were further excluded.

In the next step, we classified the rare variants according to their in silico prediction scores in Polyphen2 (http:// genetics.bwh.harvard.edu/pph2/), SIFT (http://sift.bii. astar.edu.sg/), MutationTaster (www.mutationtaster. org), CADD_phred (cadd.gs.washington.edu/) and GERP++ (UCSC Genome Browser). Taking into account variants that were present in homozygous, X-linked or compound heterozygous states, we achieved gene-based arrangements by incorporating conservation scores of the variants based on the SiPhy_29way_logOdds score.

## Validation of candidate mutations

We finally focused on variants in genes that are involved in biological pathways related to the cardiovascular system and/or in the pathogenesis of cardiac defects in animal models (http://www.informatics.jax.org/). The candidate variants were validated by Sanger sequencing (as the gold standard for screening and verifying variants of interest) for all family members to identify causative variants shared by two patients but not in the unaffected individuals. Moreover, to predict the impact of the candidate variant on protein structure and function, we also undertook protein structural modeling based on the homologous structures presente in PDB using SPDBV 4.10 (http://spdbv.vital-it.ch/). 

## Results

### Cytogenetics and Fluorescent in Situ Hybridization

None of the patients showed chromosome abnormalities in either the karyotype analysis or the FISH-based 22q11.2 microdeletion detection analysis.

### Exome sequencing and bioinformatics analyses

Five candidate variants were identified by WES (Table 1). Sanger sequencing validated the c.1583A>G (p.Tyr528Cys) variant (rs145752649) in *RASA1* in homozygous state as the only candidate variant shared by the two patients. Other family members including parents and proband’s offspring were heterozygote as expected (Fig .3). However, the mutation in *RASA1* gene has already been recorded (http://www.hgmd.cf.ac.uk/ac/index.php) in heterozygous form as the cause of autosomal dominant capillary malformation/arteriovenous malformation (CM/ AVM) (17). Moreover, as several truncating heterozygous mutations in this gene have previously been reported with vascular anomalies in association with multiple forms of CHD (18) (Table 2), we propose that the p.Tyr528Cys *homozygous* mutation could be responsible for nonsyndromic TA in our family.

### Characteristics of family members

Having evaluated the heterozygous parents more precisely, we noted a unilateral purple-red lesion (2.5×3 cm) on the father’s hand and bilateral varicose veins on mother’s legs, indicative of CM. Moreover, the father had a history of spontaneous subarachnoid hemorrhage. After the birth of the proband’s offspring, a pale-pink lesion also appeared in her forehead. None of the parents or proband’s offspring showed cardiovascular abnormality by echocardiography. The cardiologists and clinical geneticists thoroughly evaluated both patients to rule out extra-cardiac malformations. Cardiac phenotypic characterization of the patients was undertaken with echocardiography (Table 3). 

**Table 1:** Candidate variants used for cosegregation analysis


Gene	Position	Zygosity	Variant	db SNP ID	MAF in genomAD (exome_all)	CADD	GERP++	SiPhy

*RASA1*	Chr5: 86659294	Hom	NM_002890: c.1583A>G. p.Tyr528Cys	rs145752649	0.0015	28.3	5.58	15.75
*BBS12*	Chr 4: 123664906	Hom	NM_152618: c.1859 A>G: p.Gln620Arg	rs368861241	0.0005	23.7	5.81	10.955
*HUWE1*	Chr X: 53602150	Hemi	NM_031407: c. 6062C>T:p.Thr2021Ile	Novel	ND	22.6	4.37	12.15
*MYO1E*	Chr 15: 59430501	Het	NM_004998: c. 3146 C>A: p.Pro1049His	rs147579391	0.0023	31	5.79	20.044
*MYO1E*	Chr 15: 59519746	Het	NM_004998: c.554 G>A p.Asp185Gly	rs141565214	0.0022	25	6.02	16.545


Mutations were named according to http://varnomen.hgvs.org/. SNP; Single nucleotide polymorphisms, MAF; Minor allele frequency, CADD; Combined annotation dependent depletion, GERP++; Genomic evolutionary rate profiling, Chr; Chromosome, Hom; Homozygous, Hemi; Hemizygous, Het; Heterozygous, and ND; No data.

**Table 2:** Congenital Heart defects associated with CM/AVM due to heterozygous RASA1 truncating mutations


*RASA1* gene nucleotide change*	Putative effect at amino acid level	Cardiac feature

c.1572_1575dup	p.Ser526MetfsX8	CO, TOF
c.1682_1683dup	Pro562LeufsX9	CF, ASDII/PFO
c.1698+3_1698+4insT	Splicing affected	PS
c.2125C>T	p.Arg709X	CF
c.21841+1delG	Splicing affected	PDA, ASD, PS, prolapsed TV
c.806_810delTTTAC	p.Leu269ProfsX11	CO
c.957G>A	p.Trp319X	CO


*This table is adapted from Revencu et al. (18). Nucleotide numbering was based on cDNA sequence NM_002890.1. Mutations were named according to http://www.hgvs.org/mutnomen/. CM/AVM; Capillary malformation/arteriovenous malformation, CO; Cardiac overload, TOF; Tetralogy of fallot, CF; Cardiac failure, ASD; Atrial septal defect, PFO; Patent foramen ovale, PS; Pulmonary stenosis, PDA; Patent ductus arteriosus, and TV; Tricuspid valve.

**Table 3:** Cardiac phenotypic characterization of the patients


Patient no.	Cardiac phenotype	Capillary malformation symptoms

III-1	Normal values for echocardiographic measurements	A unilateral purple-red lesion (2.5×3 cm) on hand.Subarachnoid Hemorrhage
III-2	Normal values for echocardiographic measurements	Bilateral varicose veins on legs
IV-3	TA	Early onset bilateral varicose veins on legs
	Functionally single ventricle with	
	LV morphologyLV is normal with LVEF:45%	
	RV is rudimentary	
	ASD (2 cm)	
	PS	
	Small VSD	
	Mild MVP	
	Mild MR	
IV-4	TA	Early onset mild bilateral varicose veins on legs
	Functionally single ventricle with LV morphology	
	LV is normal with LVEF:45%	
	RV is rudimentary	
	ASD (2 cm)	
	PS	
	Mild MR	
V-1	Normal values for echocardiographic measurements	A pale pink lesion (2×2 cm) in forehead


TA; Tricuspid atresia, LV; Left ventricle, RV; Right ventricle, ASD; Atrial septal defect, PS; Pulmonary valve stenosis, VSD; Ventricular septal, MVP; Mitral valve prolapse, MR; Mitral regurgitation, and LVEF; Left ventricular ejection fraction.

## Discussion

Here, we report a co-segregating homozygous p.Tyr528Cys germline mutation in *RASA1* in two patients with isolated TA. *RASA1* (also known as Ras p21 protein activator 1) is a GTPase activator for normal RAS p21 but not its oncogenic counterpart. It is the first described member of Ras GTPase-activating protein (RasGAP) family that encodes a p120-RasGAP protein (16, 19). The involvement of Ras-related signaling pathways in the development of embryonic heart has been emphasized by the significant contribution of the components of these pathways in the pathogenesis of RASopathy disorders (17, 20-22). These molecular components include either RasGAP family members or other downstream molecules in the Ras/Raf/Mek/ERK cascade. Compound heterozygous missense mutations in the gene encoding NFATC1, which acts downstream of the Ras/ERK pathway, was also recently identified in a non-syndromic case of TA in a Lebanese family (1). 

In spite of the syndromic nature of Rasopathy disorders, heterozygous germline mutations in *RASA1* cause vascular development disorders without any developmental defects (23). *RASA1* haploinsufficency due to heterozygous mutations has been identified in a subset of individuals with CM/AVM (16). CM/AVM is mainly characterized by small multifocal and randomly distributed CM as pink- red to purple lesions, varicosities vein with or without deep venous anomalies, and fast flow lesions including arteriovenous malformation (AVM) or arteriovenous fistula (AVF). Vascular anomalies typically arise in several parts of body including skin, bone, muscle, spine and even brain causing life-threatening complications including bleeding and congestive heart failure (14, 15, 18, 21, 24). In our study, the daughter and parents of the proband who were heterozygous for the p.Tyr528Cys mutation displayed multiple forms of CM/AVM. The two siblings also displayed early onset bilateral varicose veins. Furthermore, these patients are at increased risk of fast blood flow lesions as their father.

Until now, more than 100 highly penetrant mutations have been identified across *RASA1* (14, 18, 25), however, no genotype–phenotype correlation has been established (25). It is noteworthy that in a study by Revencu et al. (18) several heterozygous *RASA1* mutations (mostly nonsense, frameshift and splice defect) were identified in familial cases of CM/AVM in association with multiple forms of CHD. Although They focused on various forms of vascular anomalies due to *RASA1* mutation, they did not consider the cardiac phenotypes of their patients in detail. This kind of association indicates that while there is no data suggesting that *RASA1* homozygous mutation cause more serious phenotypes, we speculate that the high mortality rate in this family along with two children affected by severe cardiac defects may be a consequence of the complete loss of the PH domain of *RASA1*.

The p120-RasGAP protein is a monomeric cytoplasmic protein with several domains (16). Each protein domain is involved in several cellular and developmental processes in a Ras-dependent or Ras-independent manner (26). In a functional study performed on homozygote mice with a point mutation in the GAP domain (Rasa1 R780Q/ R780Q), the severity of blood vascular abnormalities was identical to Rasa1-null mice and their cardiovascular manifestations were also mostly restricted to ECs. This finding suggested that cardiovascular anomalies are caused by the inability of *RASA1* to control Ras activation in a Ras-dependent manner (24). In accordance with this finding, we focused on the functional importance of the PH domain responsible for Ras-dependent function of p120RasGAP, which seems to contribute to the pathogenesis of the cardiovascular phenotype observed in the family reported here. The missense substitution p.Tyr528Cys found in our patients alters tyrosine to cysteine in the PH domain. Since this residue is extremely conserved among human PH domain-containing proteins (http://grch37. ensembl.org/index.html) and among other species, it is likely to be essential for RASA1 function.

Generally, PH domains have structurally conserved motifs and contain about 100 amino acid residues. They are present in several proteins and contribute to signal transduction pathways. The PH domain of p120RasGAP is consists of aminoacids 474-577 and is in the noncatalytic region of the protein. However, it binds to the catalytic domain (GAP domain) within the same protein and interferes with Ras/GAP interaction (27). According to Hernandez et al. (28) the PH domain of p120-RasGAP has the ability to bind to phospholipid subgroups as well as being involved in numerous protein-protein interactions. The C-terminal region of the PH domain (residues 523591) interacts with Ras and competes with it for binding to the GAP domain. The Tyr528 side chain, which is substituted by the cysteine residue in our patients, is exposed on the C-terminal region of the PH domain (27, 29). This substitution leads to the removal of the aromatic side chain and creation of a slightly negatively charged residue (28). The surface exposed position of tyrosine suggests that this substitution may alter binding of the PH domain to protein partners or the GAP domain within the same protein (29). Therefore, it seems that complete loss of function of the PH domain in a Ras-dependent manner would lead to the inability of *RASA1* to regulate the Ras molecule, and thereby may have an effect on TA pathogenesis in our patients.

Finally, we considered previous functional studies on murine models deficient for Rasa1. These data suggest the essential role of Rasa1 in the regulation of cardiovascular development. Although heterozygous mice, due to the loss of one germline Rasa1 allele, had no observable phenotype, homozygous loss caused embryonic death at E9.5 to E10.5 which is correlated with the primary formation of EC in the atrioventricular canal and outflow tract (2, 3, 19, 30, 31). Interestingly, adult mice with induced homozygous loss of Rasa1 in all tissues have no detectable spontaneous cardiovascular defect. Therefore, Rasa1 seems to be necessary for embryonic cardiovascular development, however, it is not necessary for cardiovascular maintenance (31). These studies also indicated that, while Rasa1 is ubiquitously expressed, embryonic mortality of mice deficient for Rasa1 is mostly restricted to ECs (16).

## Conclusion

On the basis of the contribution of the Ras/ERK pathway in embryonic development of heart valves, bioinformatics-based evaluation of the p.Tyr528Cys mutation, and evidence from functional studies in mice models deficient for Rasa1, we suggest that the identified p.Tyr528Cys homozygous mutation in *RASA1* is likely to be responsible for the TA phenotype observed in the pedigree analysed here. However, this hypothesis needs to be supported by generating an animal model carrying the p.Tyr528Cys point mutation. We also recommned that parents with CM/AVM should be screened for *RASA1* heterozygous mutations and if both parents are carriers, fetal echocardiography should be undertaken as a precaution in the event of pregnancy.
